# Turkey’s response to COVID-19 in terms of mental health

**DOI:** 10.1017/ipm.2020.57

**Published:** 2020-05-21

**Authors:** Hakan Öğütlü

**Affiliations:** Department of Child and Adolescent Psychiatry, Ankara City Hospital, Ankara, Turkey

**Keywords:** Coronavirus, COVID-19, pandemic, Turkey, children

## Abstract

Coronavirus disease (also known as COVID-19) continues to spread throughout the world. In Turkey, which has a strong health system, most hospitals have been turned into pandemic hospitals, elective procedures have been postponed, and doctors have been reassigned to treat COVID-19. Efforts to limit spread of COVID-19 have been effective in reducing the spread of COVID-19. Behind this success was not only the intrinsic strength of the health system but also the strict changes in everyday life wrought by the crisis. It is an inescapable fact that these new measures, such as the imposition of curfew and lockdown, have had a significant effect on the mental health of the general population. Anxiety caused by COVID-19 has spread to the mental state of everyone. Although coronavirus-related diseases will end soon, it is predicted that serious psychiatric disorders will be a lasting consequence of the pandemic. Despite the many negatives brought by COVID-19, it has led to a positive unity between the public and healthcare professionals, and in spite of significant risks to their own health, healthcare workers have risen to the challenge of COVID-19.

Coronavirus disease (also known as COVID-19) continues to spread throughout the world. In Turkey, the first case was diagnosed on March 11, 2020, a date somewhat later than other European countries. As of 28 April, 114 653 people have been diagnosed as COVID-19 positive and 2992 people have lost their lives. About 1621 patients have been treated in an intensive care unit (ICU). The mortality rate due to COVID-19 was 2.6% in the country (Ministry of Health, 2020). In Turkey, which has a strong health system, most hospitals have been turned into pandemic hospitals, elective procedures have been postponed, and doctors have been reassigned to treat COVID-19. The first case was detected late due to the strict supervision of people who came from abroad. A policy was quickly established for COVID-19 treatment. The ICU bed number was approximately 32 000, a figure deemed sufficient for the country’s needs. For these reasons, there was no period when resources were exhausted or when health services could not be offered due to COVID-19. Even in Istanbul, where the virus was most intense, the occupancy rate for ICUs was 59.5%, while the maximum bed occupancy rate was 50.2% by April 10 (Habertürk, 2020) . Moreover, in recent days, there has been a decrease in the number of new cases (BBC Turkish, 2020). Of course, behind this success was not only the intrinsic strength of the health system but also the strict changes to everyday life necessitated by the pandemic.

A scientific committee was set up to establish COVID-19 precautions in Turkey. Schools and universities were closed on March 12, the day after the first case was reported. Online education for students was quickly put in place. On March 13, transportation restrictions, including the closing of borders and halting of flights began and quarantine measures for people returning from other countries, were applied. Two days later, public places such as restaurants, cafes, bars, libraries, and places of worship were closed. On March 21, 10 days after the initial case, a curfew was imposed on people over the age of 65 and those suffering from chronic illnesses. On April 3, a partial curfew was extended to cover those under the age of 20, and it became mandatory for all citizens to wear masks in public places. Finally, on April 11, a general curfew was announced, covering everyone at weekends; this practice will continue for the foreseeable future (Anadolu Agency, 2020). What measures are taken beyond that are, as yet, unknown. However, it is an inescapable fact that such drastic change in people’s lives will have a significant effect on the mental health of the general population.

When the virus started in China, the public thought that it would be limited to China. As the virus spread to other countries, there were claims on social media that the virus would not come to Turkey and that even if it did, there would be no transmission due to genetics (Sabah, 2020). These misguided claims prevented people from preparing adequately for the effects of the virus. Moreover, the rapid spread of the virus in Europe began to cause anxiety and shopping for cleaning supplies and basic foodstuffs quickly increased. With the outbreak of the virus in the country, extreme scenes were witnessed in pharmacies and grocery stores as an air of panic took hold. Protective face masks, medicines, food supplements, and cleaning supplies were all well stocked and stores never ran out of necessary supplies. Again due to excessive speculation on social media, excessive anxiety took hold among the general public.

Before the outbreak, Turkey had traditionally high standards of hygiene practices. The high tendency to hand wash among people was both cultural and religious in origin. The cultural behaviors of Turks on cleaning date back to the Anatolian Seljuk and Ottoman periods. The pursuit of cleanliness, which was prioritized by Islam, led to the reputation of Turkish baths (Disli & Ozcan, 2014). Besides, the historical curative and sanitizing effects of vinegar were introduced during periods of plague and epidemics (Bulmuş, 2012). The plague treatment included prescriptions using vinegar in Ottomans. Great attention was taken to the use of vinegar in cleaning traditionally; vinegar was declared a piece of cleaning equipment. Everything from outside the house was kept in water with vinegar or was taken home after waiting for one day on the balcony as a precaution. As a disinfectant, cologne (kolonya), one of Turkey’s national symbols, was in great demand. European cologne was first used in the Ottoman Empire during the reign of II. Abdulhamid (1870–1909) had been a part of daily cultural life with its sanitizing and perfume features (Yentürk & Yentürk, 2001). The cologne in every Turkish home was served as a tradition to cool guests. Since cologne contains 70% alcohol, an accepted virus-killing agent, it was stocked in every home (BBC Travel, 2020). With the recommendation of the World Health Organization that the use of masks should only be used to prevent the ill person from infecting others, masks have become an indispensable part of life in Turkey. Some people even began to sew their own masks. This problem was solved when the government began distributing free five masks per week to each person. It also became unacceptable for people to enter indoor spaces without a mask.

The most encouraging part of this process was the public applause to support frontline health workers from balconies every evening at 21:00. This communal expression of support made healthcare workers extremely proud of their profession. While all other employees were given the message ‘Stay Home’, the applause was a great boost during this period when healthcare professionals had no choice but to work hard in demanding conditions. Health workers also emphasized the importance of social isolation by sending posts urging the public to stay at home. Over time, this practice has evolved such that 21:00 now marks a time of celebration and fun in every home.

During the lockdown, people began to experience tension in their inter-personal relationships due to increased anxiety. Anecdotally, a range of different behaviors have been reported as people try to adapt to the new conditions such as communication problems between spouses, tension between parents and children, increased irritability and anger outbursts, depressive and anxious mood, excessive use of social media, and binge eating. With a lack of opportunities to engage in social activities outside the home, families have been forced to spend more time enclosed leading to increased tension and acrimony in the home. On the other hand, the fear of parents of infecting their children with COVID-19 has had negative effects on them, leading them to take extra precautions with their children. Children were kept inside, often against their wishes, and were not able to communicate with their friends and relatives. Children were no longer given access to their typical social support networks.

After the closure of the schools, children could not envisage the full extent of the situation and saw it as a holiday from school and an opportunity to spend more time with their families. However, fears began to emerge that COVID-19 could kill their parents or close relatives. In addition, parents behaved differently and more inconsistently than usual due to elevated stress levels invoking confusion among the children who expected more attention from them. Mental health professionals, recognizing the fraught nature of the new conditions, developed materials for parents and their children and made them available free of charge. These psychoeducation materials were mostly made up of drawings, stories, and cartoons. Furthermore, they broadcasted live on social media in an effort to prevent misinformation from less reliable sources, helping people to solve these problems as well as offering advice on how they should continue their lives under lockdown. Increases in similar problems created a greater demand for online therapy. In order to avoid people having to attend hospital in person, telepsychiatry services were offered to people who required such intervention.

The psychological toll on frontline healthcare workers, directly exposed to the burden of the pandemic, became even more imperative. On returning home after work, they could not allow their children give them a loving hug and had a difficult time explaining to them why they could not do so (Medimagazin, 2020). Additionally, after encountering very sick patients, healthcare workers also experienced an acute stress response at home, easily becoming angry, emotional, and behaving erratically. Sleeping and eating patterns also became significantly disturbed. The possibility of future post-traumatic stress disorder is also something that healthcare workers must contend with.

The effect of the pandemic on children has also been acute: terrified of COVID-19 infecting their family, and the possibility of their parents dying, children acted out and began exhibiting more oppositional behaviors. Again, mental health professionals recognized the difficulties being faced in families, first offering their expertise and support to healthcare workers and their children. Following this initiative, both psychiatrists and psychologists began to offer therapy free of charge, allowing healthcare professionals only to make a telephone appointment. The Ministry of Health has also developed unique software to allow mental health professionals to provide support to healthcare workers and their children (Ministry of Health Health Services General Directorate, 2020). Moreover, professional associations have developed a fairy tale reading project in order to support children. Celebrities and relatives of children who were not physically in contact with their parents were asked to read and record fairy tales for the children of healthcare professionals. The children started to listen to these tales every evening before going to bed, so they could feel the support of the people they wanted to hug and embrace (Turkey Association for Child and Adolescent Psychiatry, 2020).

One of the biggest pandemics in the history of Turkey was the cholera pandemic, first seen in 1822, and which continued in different cities from time to time until 1910 during the rule of the Ottoman Empire. In order to prevent the spread of cholera, the Ottomans set up teams to oversee cleanliness in cities by keeping hygiene at the forefront. They distributed brochures to inform the public about the disease and the necessary precautions to prevent its spread. Clerics (Imams) preached about cholera to the community in mosques. Provincial borders were closed and those wishing to enter cities were quarantined for at least three days (Gökçe, 2001; Yılmaz, 2017; Bolaños, 2019). In this period, when modern psychiatry was not yet developed, the cholera epidemic was brought to an end after the necessary precautions were taken and treatments were applied. However, sufficient information about the psychiatric sequelae arising from this pandemic was not recorded in the literature.

In the current crisis, although taking similar measures to the Ottomans, the faster and more effectively they are imposed has resulted in reducing the local spread of the virus. With the advent of globalization, not only has COVID-19 spread faster than all previous diseases, but COVID-19 anxiety has also infected the mental state of populations all over the world. Although coronavirus-related diseases will end soon, it is predicted that serious psychiatric disorders will be a lasting legacy of the pandemic. Modern psychiatry must address the psychiatry of pandemics as a matter of urgency.

Therapy services in child psychiatry and in adult psychiatry during the present crisis have been temporarily postponed, while inpatient and outpatient services continue with greatly reduced numbers. The reasons for this reduction were that many people avoided hospitals to reduce transmission and a number of mental health professionals were reassigned to work in outpatient COVID-19 clinics and services. Our colleagues, who experienced great panic at the beginning of the crisis due to the onerous responsibility brought on by the state of emergency, have been proud to serve COVID-19 patients by adapting to the process over time and taking the necessary protection measures. At the end of this pandemic and beyond, mental health services will be in great demand. By giving priority to these services, it will be essential to provide psychiatric support to all of those in need, especially to healthcare professionals working on the frontline. For this to happen, it is essential to ensure the continuity of the government institutions and voluntary initiatives already established. Despite the many negative effects of COVID-19, it has led to a greater unity of purpose between healthcare professionals and the general public. Given the risks to healthcare professionals’ own health at this time, a huge debt of gratitude is owed to the healthcare professionals working on the front line during this hugely challenging period.
